# Microglial dynamics and neuroinflammation in prodromal and early Parkinson’s disease

**DOI:** 10.1186/s12974-025-03462-y

**Published:** 2025-05-21

**Authors:** Frida Lind-Holm Mogensen, Philip Seibler, Anne Grünewald, Alessandro Michelucci

**Affiliations:** 1 Neuro-Immunology Group, Department of Cancer Research, Luxembourg Institute of Health, 6A, rue Nicolas-Ernest Barblé, Luxembourg, L-1210 Luxembourg; 2 Faculty of Science, Technology and Medicine, University of Luxembourg, 2, avenue de l’Université, Esch-sur-Alzette, L-4365 Luxembourg; 3 Institute of Neurogenetics, University of Lübeck and University Hospital Schleswig-Holstein, Ratzeburger Allee 160, Lübeck, 23562 Germany; 4 Luxembourg Centre for Systems Biomedicine, University of Luxembourg, 6, avenue du Swing, Belvaux, L-4367 Luxembourg

**Keywords:** Microglia, Neuroinflammation, Parkinson’s disease, Prodromal stage, REM-sleep behaviour disorder

## Abstract

Parkinson’s disease (PD) is characterized by a drastic loss of dopaminergic neurons already at diagnosis. As this loss of neurons starts decades before diagnosis, understanding the prodromal stages of the disease might offer novel strategies to curb its progression. While the precise pathogenic mechanisms underlying PD remain incompletely understood, growing evidence suggests that neuroinflammation and immune dysregulation play a central role in the development and progression of the disease. Here, we delve into the emerging roles of microglia, the resident immune cells of the central nervous system, in the pathogenesis of prodromal and early-stage PD. We emphasize that microglia contribute to neuroinflammation, protein aggregation and neurodegeneration, although the underlying mechanisms are not yet known. Neuroimaging studies have provided valuable insights into the patterns of microglial activation detected in individuals with prodromal PD and at the time of clinical diagnosis. Furthermore, we highlight the complex interplay between immune dysregulation and neurodegeneration along PD development, including alterations in the peripheral immune system, brain-gut interactions and brain-immune interfaces. Lastly, we outline existing models for investigating microglial involvement in prodromal PD, along with the impact of anti-inflammatory therapies and strategies to modify risk factors. In conclusion, targeting microglial activation and immune dysfunctions in individuals at risk of PD could represent a promising preventive measure and may offer novel therapeutic strategies for early intervention and disease modification.

## Introduction

Parkinson’s disease (PD) is the fastest-growing neurological disease worldwide [32]. Around 10–15% of patients have a family history of PD with a direct genetic cause, whereas recent studies found that risk variants could explain up to 30% of the heritable risk of PD depending on prevalence [102]. Rare mutations in several genes have been detected to cause autosomal dominant or recessive monogenic forms of PD. Some of these genes, including *LRRK2*, *SNCA* (which encodes α-synuclein), *PRKN, PARK7* and *PINK1*, encode proteins involved in immune functions [142]. However, the large majority of PD patients are idiopathic.

**Fig. 1:**
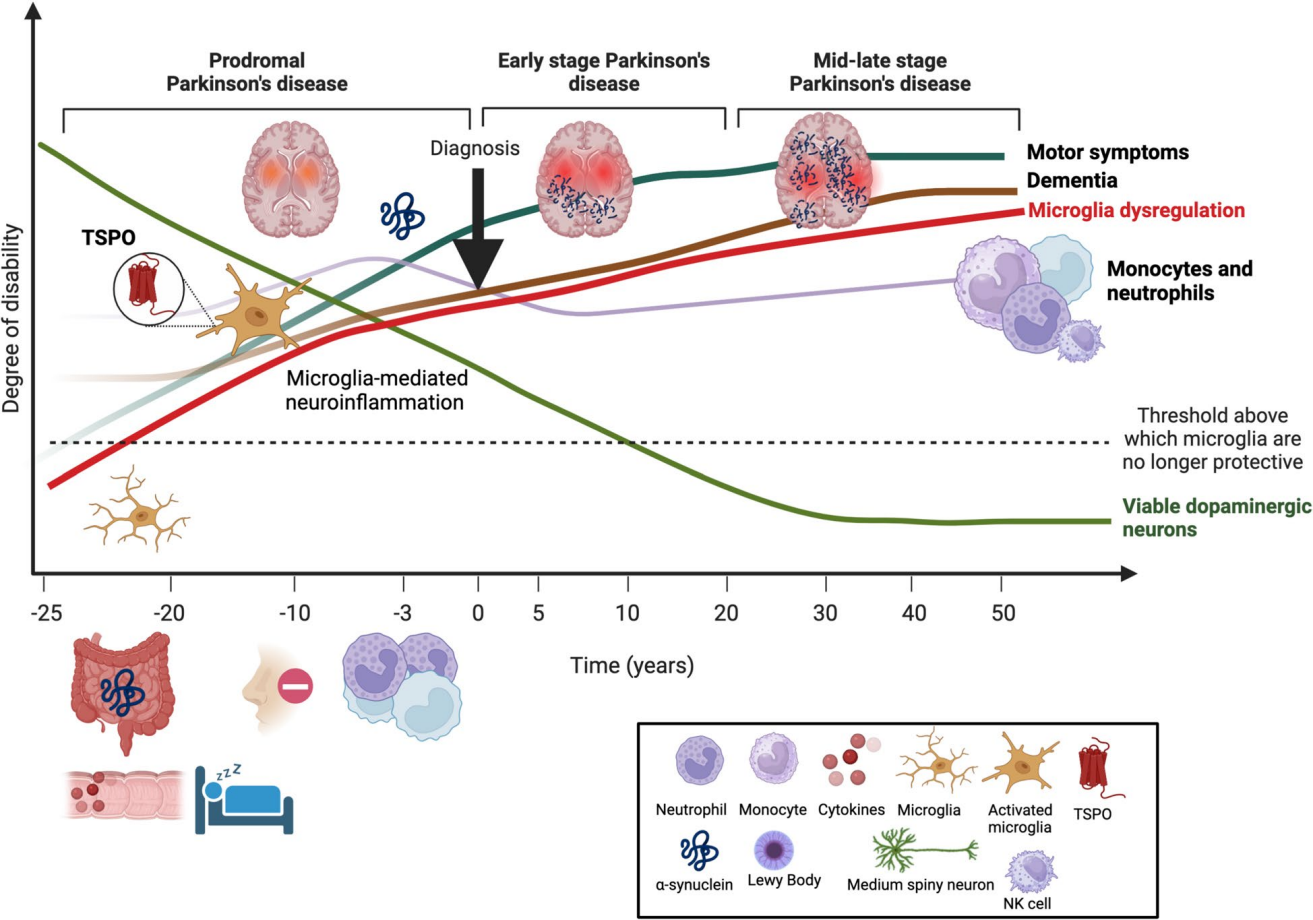
Microglia and immune system changes along PD trajectories. On the y-axis, the degree of disability is plotted. The x-axis depicts the time in years from 25 years before diagnosis until the end of life of the patient. Square brackets represent different stages of the disease. Coloured lines represent different symptoms or cellular changes. Individuals suffer from constipation up to two decades before diagnosis. Hyposmia is commonly present a decade before disease diagnosis. Approximately 80% of RBD patients eventually develop PD with a delay of approximately 10 years - a subset of these show microglia activation. At diagnosis (black arrow), around 50–70% of viable dopaminergic neurons are lost, while α-synuclein accumulation and Lewy bodies are found post-mortem, but are believed to be present at disease diagnosis. The entity of dementia (brown line) and motor symptoms (dark green line) increase with disease duration

Motor impairments, including bradykinesia, tremor and rigidity, characterize PD patients. These symptoms represent the pathological consequences of the degeneration of dopaminergic neurons in the midbrain. The hallmark neuropathological characteristic of PD is the accumulation of Lewy bodies, which are composed of fibrillar α-synuclein and ubiquitinated proteins, within the neurons of the *substantia nigra*. Prior and alongside the appearance of motor signs, patients present with non-motor symptoms, such as constipation, olfactory dysfunction, sleep and mood disturbances [1, 33, 88, 103]. According to clinical evidence and symptoms, PD patients have been categorized to fall into three stages, namely the preclinical, prodromal and motor/clinical phases [136]. In the preclinical phase, individuals may not exhibit clinical signs or symptoms, thus PD-specific pathology is based on genetic, molecular and imaging biomarkers [9, 88]. Patients exhibiting non-motor symptoms without motor signs do not meet the diagnostic criteria for a PD diagnosis, therefore they are classified as prodromal PD patients. This prodromal stage can last years to decades before an identifiable PD diagnosis (Fig. 1) [118]. One notable predictor of a PD diagnosis is rapid eye movement (REM) sleep behaviour disorder (RBD) with up to 80% of RBD sufferers eventually developing PD [8, 113]. RBD can be present up to 20 years prior to initial PD diagnosis.

Currently, both clinical non-motor markers and nonclinical biomarkers define the research criteria for prodromal PD, with varying levels of certainty. Here, we are particularly interested in these early phases as the neuronal demise might still be preventable. At diagnosis, at least 40–60% of dopaminergic neurons are already lost and the disease has progressed too far for recovering using any therapeutic intervention [37, 47] (Fig. 1). However, focusing on individuals yet in the early stages presents a potential window of opportunity for preventive measures, disease-modifying interventions and therapeutics. Critically, for personalized prospects, it should be taken into consideration that prodromal phases of PD are heterogeneous and symptoms can vary significantly between individuals, regardless of whether the case is idiopathic or familial [5].

Microglia, the immune effector cells of the central nervous system (CNS), are the first brain parenchymal cells to respond to threatening conditions, oxidative stress or infections, making them essential targets to study when seeking early indicators of CNS dysregulation. Microglia have emerged as key players in the pathogenesis of neurodegenerative diseases, including PD, contributing to neuroprotection, neuroinflammation and neurodegeneration [6]. While microglial activation has been observed in patients with PD and Alzheimer’s disease (AD) at late stages and in post-mortem tissues [40, 44, 63, 95, 129], their specific role in prodromal and early-stage PD remains largely unknown. Until recently, most studies examining immune changes focused on patients with established locomotor disabilities, limiting insights into the temporal relationship between immune alterations and the onset or progression of motor symptoms (Fig. 1). However, a clearer understanding of the immune system’s role in PD is now emerging from studies involving individuals at high risk of developing PD or related synucleinopathies, who may be in the prodromal stage of the disease and that could aid in the diagnosis. A recent bibliometric study found a consistent increase in the number of publications on prodromal PD since 2017, indicating that PD precursors are gaining more attention and their analysis is rapidly increasing [156].

In this review article, we strive to highlight the importance of studying the role of microglia and immune dysfunctions in prodromal and early-stage PD, including changes in microglial activity from neuroimaging studies, alterations in peripheral blood-immune system, brain-gut interactions and brain-immune interfaces. Lastly, we describe current models to study microglial cells in prodromal PD as well as the effects of anti-inflammatory treatments and risk factor modification. Identifying microglial cell changes and immune dysfunctions in prodromal and early-stage PD would contribute to determining the underlying mechanisms of the disease and the prospective therapeutics that would ideally counteract the disease trajectories by modifying central and peripheral immune cell phenotypic states supporting these paths.

## Microglial cell changes in PD patients

### Microglial cell phenotypic adaptations and heterogeneity

Although microglia can have neuroprotective functions in the CNS, pathological insults, such as α-synuclein accumulation, Lewy body formation, and dying neurons over time activate microglia, which secrete pro-inflammatory cytokines and alter their neurotrophic functions creating a neurotoxic environment [38, 49, 162]. Increased numbers of microglia have been observed in the *substantia nigra* (SN) of PD brains together with the acquisition of an activated amoeboid morphology, in both SN, amygdala and hippocampus (HC) of PD patients and incidental Lewy body dementia [72]. Interestingly, this occurred irrespective of α-synuclein pathology [31]. Similar observations were recapitulated in a mouse model of preformed α-synuclein fibrils injection, where microgliosis occurred in early stages of neurodegeneration and in other brain regions distant to injection and did not correlate with α-synuclein load [42]. These results indicate that microglial cells morphologically and molecularly adapt prior accumulation and aggregation of α-synuclein, thus suggesting that other cues present in the SN and HC can affect their phenotypic acquisition. Hence, these observations challenge the hypothesis of an early microglial activation in response to α-synuclein aggregates and neurodegeneration, leading to an alternative view where microglia play already a causal role during prodromal PD independently from α-synuclein accumulation. Genetic risk factors and environmental stressors can disrupt microglial homeostatic functions, shifting them from homeostatic and neurotrophic cells towards harmful pathogenic phenotypes. These defective microglial cells can exhibit exhaustion, premature senescence, impaired phagocytosis and lysosomal dysfunction. As such, they possibly create a self-perpetuating cycle of α-synuclein accumulation, mitochondrial impairment, chronic neuroinflammation, and synaptic dysfunction that precedes overt neurodegeneration. Targeting microglial homeostasis, metabolism and early dysfunctional cells during this window, for example by enhancing mitochondrial function or autophagy, could delay or prevent clinical PD onset [89]. On the other side, microglia can also take on protective roles in PD, for example by taking up α-synuclein aggregates from overloaded neurons and by providing functional mitochondria via tunnelling nanotubes rescuing neurons from oxidative stress and mitochondrial dysfunction [19, 120]. Lastly, the heterogeneity and the dynamics of microglial cell substates should also be taken into account. Single nuclei RNA sequencing of post-mortem SN tissue identified various subtypes of microglia, where some cells express genes related to protective functions and others contribute to neurodegeneration, with the proportions of protective subpopulations being disturbed in PD [91, 129]. Furthermore, the expression levels of various genes involved in dopamine metabolism are modulated in PD, including a significant decrease of tyrosine hydroxylase expression not only in neurons, but also in glial cells in the SN of PD patients [91]. However, a deep characterization of microglial subpopulations in prodromal and early PD patients has not yet been conducted, mainly due to the invasive nature and ethical constraints of obtaining midbrain biopsies from those individuals.

### Live imaging analysis of microglial cell modifications

In vivo assessment of microglia is possible by positron emission tomography (PET) and single-photon emission computed tomography (SPECT) imaging employing specific ligands targeting microglial activation markers and allowing spatially resolved quantification of neuroinflammation, distinguishing brain regions like the SN, which is central to PD pathology. One of them is the 18 kDa Translocator Protein (TSPO), which is barely expressed in the healthy brain and highly upregulated in the outer mitochondrial membrane of activated microglia [85, 108]. Increased TSPO binding has been observed in multiple brain regions, including the SN and basal ganglia correlating with disease severity and progression [44, 61]. Interestingly, microglia activation has been detected in the SN, anterior cingulate cortex and prefrontal cortex of PD patients in the earliest stages of the disease, between 7 and 12 months after diagnosis. These patients also accumulated high levels of carbonylated proteins in the cerebrospinal fluid (CSF), a sign of oxidative stress alongside neurodegeneration and microgliosis [61]. Compelling evidence of microglial activation in prodromal PD patients has been reported in a case-control study of 20 RBD patients, who exhibited microglial activation and decreased dopaminergic neuronal function in the SN [138]. A follow-up study conducted on a subset of these patients revealed a progressively decreasing function of the nigrostriatal pathway, which was even more pronounced in patients showing an elevated baseline microglial activation in these areas [135]. This suggests that in these patients, microgliosis may have a detrimental role in prodromal PD [134]. However, TSPO is also expressed in other cell types involved in brain inflammation, such as astrocytes and infiltrating immune cells, which can complicate interpretation [77]. The ligand ^11^C-R-PK11195 (PK), binding TSPO, is used as a marker for ongoing neuroinflammation in patients, but suffers from low signal-to-noise ratio and non-specific binding, limiting its sensitivity. Advanced tracers, such as [^18^F]DPA-714, have shown consistent results in preclinical models, correlating cerebral inflammation with motor impairments in PD rodent models, but require further validation for widespread clinical use [105]. Taken together, despite promising reproducibility in animal models and early human studies, continued development of advanced radiotracers and standardized protocols is essential to improve their clinical utility.

## Modifications of the peripheral immune system in prodromal PD patients

PD is nowadays increasingly recognized as a systemic disorder with evidences of molecular and cellular alterations in both the CNS and peripheral immune systems. Therefore, analyzing peripheral immunity may provide valuable insights and serve as a more accessible proxy for monitoring disease-related changes that are otherwise challenging to assess directly in the brain. In this context, a recent study investigated peripheral blood immune system changes via DNA methylation deconvolution in PD and prodromal PD patients and found many similarities in innate immune changes between the two groups [112]. Another recent work investigated the composition, gene expression and inflammatory response of peripheral blood mononuclear cells (PBMCs) to α-synuclein oligomers and monomers previously found in the plasma of PD patients. The study showed that healthy PBMCs reacted to both monomeric and oligomeric α-synuclein by elevating cytokine and chemokine release, but PBMCs from PD patients did not respond and had a low rate of internalization of α-synuclein [151]. These results are in line with the hyporesponsive phenotype identified in bone marrow-derived macrophages (BMDMs) from a DJ-1 KO mouse model [84]. The same PBMC study found correlations between subsets of natural killer (NK) cells and monocytes and the PD subtype. Patients with olfactory deficits exhibited a lower percentage of classical monocytes, while PD patients with constipation had a lower percentage of mature NK cells together with a higher percentage of unconventional NK cells [151]. These results show how peripheral immune markers could help in patient stratification and detection of early-stage PD. Regarding the changes in the adaptive immune cells, the modifications observed in prodromal PD did not follow completely what was found in established PD and these perturbations occurred at slightly different periods compared to innate immune perturbations. Most strikingly was a significant increase in CD4+ naive cells after diagnosis [112]. While beyond the scope of this review, peripheral immune changes in prodromal PD have been recently reviewed [145].

In RBD individuals, a prospective case-control report detected a higher percentage of classical monocytes and mature NK cells in those patients with significantly elevated PK binding and reduced putaminal dopaminergic function compared to healthy controls [36]. Monocytes from RBD patients showed increased levels of integrin alpha M/cluster of differentiation 11b (CD11b) and toll like receptor 4 (TLR4) expression together with decreased levels of HLA-DR compared to healthy controls. Interestingly, a putatively protective cell subset of CD163^+^ classical monocytes has been detected in RBD patients. This subset correlated with lower levels of microgliosis in the SN and higher dopamine in the putamen compared with other RBD patients without this subset. On the contrary, TLR4 expression on monocytes was positively correlated with microglia activation in the SN (measured by ^11^C-PK11195 PET tracer) and lower putaminal dopaminergic function (determined via ^18^F-DOPA PET) in RBD patients [36]. This study uncovered an important role for monocytes in RBD patients in driving the disease process and places the monocytic population as a possible biomarker and potential target. However, to better understand whether changes in the peripheral immune system during prodromal PD mirror processes occurring in the brain, a thorough characterization is warranted (See Table 1. Outstanding questions). This could help determine if cellular ratios or markers obtained through less invasive methods, such as blood tests, could serve as valuable diagnostic or prognostic biomarkers.

**Table 1:** Outstanding questions

A.* Research-oriented queries*
1. *Does neurodegeneration cause neuroinflammation or does neuroinflammation drive neurodegeneration? Focuses on mechanistic causality rather than direct applications.*
2*. Is the dysregulation of microglia seen in prodromal PD intrinsic to microglia or is it induced by cues from the CNS or even from the peripheral nervous or immune systems? Examines fundamental microglial biology.*
3*. Are some forms of PD (iPD, LRRK2, PARK7) more microglia-mediated than others? Compares disease subtypes’ pathobiology.*
4*. Are PD mouse models comparable to patients when studying the prodromal and early PD stages? Evaluates model systems’ translational validity.*
5.* Is the impairment in glymphatic activity in Parkinson’s patients a consequence of sleep disturbances or vice versa? Investigates pathophysiology of sleep-related symptoms.*
B.* Clinical-oriented queries*
1*. What drives disease progression in the prodromal phase? Links to biomarker development and neuroprotective trial design.*
2*. How do we improve the assessment of early microglia-specific changes in PD patients? Could for example liquid biopsies (plasma, CSF, extracellular vesicles) inform on microglial functions? Addresses diagnostic tool development also for patient stratification.*
3.* By halting microglial changes, could neurodegeneration be reversed or decreased? Tests therapeutic strategies in trials.*
4*. Should neurologists already advice life-style changes to RBD patients or prodromal PD patients, such as more hours of sleep and more exercise? Supports current lifestyle interventions for risk reduction.*

## The brain-first versus body-first hypothesis

### Body-first type of PD

The brain-first versus body-first hypothesis in PD suggests that α-synuclein pathology may originate either in the brain (e.g., olfactory bulb or amygdala) or in the body (e.g., enteric nervous system), spreading via neural connections to other regions. In the body-first type of PD, patients are less likely to have mutations leading to or increasing the risk of PD and they are RBD-positive in the prodromal phase. In the brain-first PD form, patients are more likely to harbour mutations and they do not present with RBD in the prodromal stage [11]. Interestingly, two reports showed a higher percentage of patients with RBD, which were positive for pathological α-synuclein aggregates in colonic biopsies (64% and 24% respectively) compared to patients without RBD or unknown RBD status (13 to 5% respectively) [78, 133]. Intriguingly, α-synuclein accumulation was found in submandibular gland biopsies of 92% of PD patients with a history of RBD and only in 55% of patients without RBD [152], pointing towards an α-synuclein pathology present in the peripheral nervous system more frequently in patients with RBD. Furthermore, submandibular gland biopsies were proposed to be a promising and safer procedure to diagnose prodromal PD in RBD patients and to confirm a PD diagnosis [152].

Constipation is a common prodromal PD symptom and has a positive likelihood of 2.5 for the development of PD [53]. However, the predictive value of constipation is moderate with a high sensitivity but low specificity, as it represents a common feature in the general population. A case-control study showed that constipation could occur up to 20 years before diagnosis [119]. Prodromal constipation might mark the body-first PD subtype, which starts in the enteric nervous system [11]. According to the definition of this subtype, α-synuclein aggregates are first observed in the enteric nerve terminals or other peripheral nerves and spread via the pons to the midbrain, thereby also affecting non-dopaminergic neurons in the raphe area and locus coeruleus (LC) prior to affecting the SN [13]. Throughout, α-synuclein aggregation and accumulation have been detected in the neurons of the intestinal tissue of both prodromal and symptomatic PD patients [137].

### Microbiota-driven gut inflammation

Gut inflammation and dysbiosis are common traits in both manifested and prodromal PD patients, indicated by elevated levels of calprotectin, IL-1α, CXCL8 and C-reactive protein in stool samples [58, 122]. Intriguingly, large cohort studies have demonstrated a link between PD and inflammatory bowel diseases (IBDs), including Crohn’s disease, with IBD patients showing an increased risk of developing PD [153]. Furthermore, variants of the *LRRK2* gene have been associated with increased susceptibility to both PD and Crohn’s disease [65]. Therefore, gut inflammation and higher LRRK2 levels in Crohn’s disease may be biomarkers of increased risk for idiopathic PD and could even represent tractable therapeutic targets [55].

Interestingly, recent studies found altered gut microbiota in early PD and RBD patients compared to healthy controls and first-degree relatives of RBD patients, suggesting that gut dysbiosis occurs already at the early stages of PD and in RBD [59]. One of the main differences in the composition of the gut microbiome in prodromal PD patients (RBD positive patients), is a prominent decrease in short chain fatty acid producing bacteria compared to healthy controls [59]. Nonetheless, in the newest MDS criteria for prodromal PD, gastrointestinal dysfunction is still considered a “promising candidate marker”, due to potential surveillance bias in analyses [53]. Further longitudinal studies encompassing pre-clinical and prodromal PD to manifesting and late-stage PD are needed to investigate whether dysregulated gut microbiota composition and intestinal permeability may contribute to peripheral immune dysfunction and neuroinflammation via the gut-brain axis as a part of PD pathogenesis [86]. This axis represents an important regulator of glial functions. For example, under homeostatic conditions, the gut microbiome regulates microglial maturation and activation via the release of short-chain fatty acids [34]. During aging, metabolites derived from the gut microbiota also function in triggering microglial cell death [144].

## The “infection hypothesis”

Since the influenza pandemic in 1918, the first cases of secondary Parkinsonism were observed and theories emerged claiming that viral infections and bacterial meningitis could in some cases result in PD [54]. The “infection hypothesis” postulates that infections drive the system towards the onset of neurodegenerative diseases, including AD or PD, by either causing direct harm via an infectious agent or by triggering neuroinflammation [12, 68]. Microbes can evade the immune system and cause latent infections that then can flare up and cause cerebral inflammation, in the end increasing the risk of developing PD. Interestingly, studies found a 1.17–1.5 fold increased risk of PD in individuals infected with herpes simplex virus (HSV-1) or varicella zoster virus [22, 74]. Other studies identified elevated HSV-1 antibody levels in both the bloodstream and CSF of PD patients relative to healthy individuals [92–94]. A recent study found an elevated risk of developing PD in individuals that were infected with an influenza virus 5 to 10 years prior to diagnosis [82]. Similar risk associations were found in a case-control study where influenza was linked to PD diagnosis more than a decade after infection [24], hinting towards infections playing a role in driving the disease process in the prodromal disease stage. Case studies of patients presenting with parkinsonism after infection with severe acute respiratory stress syndrome coronavirus 2 (SARS-COV-2), as well as studies of exacerbating cellular pathology and symptoms in mouse models [79, 130], sparked the interest in investigating whether SARS-COV-2 infections could potentiate or lead to PD. However, there is still a critical lack of evidence, mainly due to the short study timeframe considered when analysing whether SARS-COV-2 infection can increase PD incidence [98]. Of note, with vaccines already existing for certain implicated viruses, immunization could serve as a preventive strategy to lower the risk of PD.

*Helicobacter pylori* infections and periodontal bacteria have been shown to increase the risk of PD and have been linked with neuropathology without a known direct entrance to the CNS [110]. Pieces of evidence now suggest that pathogens can either enter the CNS directly across the blood-brain barrier (BBB) or secondarily initiate neuroinflammation via extracellular vesicles, lipid mediators or cytokines [17]. A study including more than three million individuals from the Swedish Total Population Registry, found that hospital-treated gastrointestinal and respiratory infections increased the risk of developing PD if the infection occurred at the age of 21–30. The study used sibling comparison to control for shared genetic and environmental factors [154]. Others found an increased risk of PD after gastrointestinal infections in men and women above 70 years of age [104].

### The “endotoxin theory”

Within the “infection hypothesis”, the so-called “endotoxin theory” states that parts of the gram-negative bacterial cell wall can play an important role in the pathogenesis of PD. Lipopolysaccharides (LPS) in the blood can be seen as a sign of systemic low-grade inflammation, which can also influence the brain [14]. Recent studies investigated previously described links between elevated LPS-binding protein (LBP) in the blood of PD patients and found that LBP levels were higher in prospective PD cases (an average of 8 years before diagnosis) and increased the risk for PD incidence, especially in women and obese individuals [164]. Interestingly, elevated LBP blood levels also increased the risk of Crohn’s disease [75]. It is clear from multiple studies using rodent models that various doses of LPS injected in the periphery can induce neuroinflammation 132]. Interestingly, a single high dose of LPS can induce neurodegeneration [116]. These molecular studies, together with the observed symptoms, such as immobility, anxiety and depression, resulted in the use of LPS as a model of brain inflammation in PD [29]. Among the danger-associated molecular patterns (DAMPs) that activate microglia in PD, α-synuclein acts through TLR4 and TLR2 binding [39]. Gut dysbiosis, especially if favouring gram negative LPS producing bacteria, could trigger initial PD pathology in the intestinal tract via TLR4, as shown in a proof-of-concept study by Perez-Pardo and colleagues [109]. Interestingly, increased intestinal permeability in early PD patients correlates with increased intestinal mucosa staining for *E. coli* bacteria, nitrotyrosine, and α-synuclein as well as serum LBP levels [41]. However, few studies so far investigated LPS and LBP levels in the blood of prodromal PD patients or during longitudinal studies from pre-clinical PD to late PD, which will be important to assess the correlation between, inflammation, neuroinflammation and PD symptoms.

### Infections and α-synuclein

Several reports suggested that α-synuclein plays a direct role in clearing infections by acting as an alarmin [52], as monomeric α-synuclein could be induced during various immune responses and protects against pathogens [66]. However, few studies from patients show α-synuclein aggregates caused by a viral infection. This was shown in the SN of patients infected with human immunodeficiency virus [69] and in duodenal tissue from children with norovirus. The α-synuclein aggregates were present in neuronal processes and the number of aggregates increased with the degree of inflammation [139]. Furthermore, studies from mouse models showed α-synuclein aggregates after viral infections. One report found α-synuclein in the gray matter of West Nile Virus-infected mice and found that α-synuclein restricted virus infection of neurons [7]. Another study found that infection of mice with H1 N1, a strain of influenza A virus, induces seeds of aggregated α-synuclein in infected neurons [90]. The aberrant proteostasis induced by viral infections may be an additional causative factor initiating protein misfolding. However, despite several correlative evidences, there is a noticeable lack of confirmation showing a direct link between infections and the increase of PD risk or exacerbation of PD symptoms. The exclusion of patients with systemic or CNS infections in relation to neurodegenerative disease studies represents a complication in conducting these analyses. This is especially important when investigating patients at risk for developing PD, such as potential prodromal PD patients, as infections may directly increase the risk of developing PD or speed up the progression. However, common infections, such as cytomegalovirus and Epstein-Barr virus, which are present in a large percentage of the world’s population, recently gained attention [15, 158]. These stealth pathogens or quiescent infections are latent, but could mediate bystander damage or activation of microglia or peripheral immune cells, flare up and re-infect [81]. Future studies should take into account this dimension and investigate in large prospective cohorts of clinically healthy individuals and diagnosed PD patients, whether infections could increase the risk or speed up the progression of PD. In addition, investigations of whether polygenic risk scores and specific genetic variants of PD could make individuals more susceptible to infections or whether infections could potentiate PD symptoms in patients with a specific genetic background are warranted (see Table 1. Outstanding questions).

## Changes in brain-immune interfaces in prodromal and early-stage PD

Threatening conditions throughout the body, such as infections, can lead to impairments at key brain immune-interfaces, including the BBB, the blood-cerebrospinal fluid (B-CSF) barrier, the glymphatic and meningeal lymphatic systems, which are compromised in PD patients. Dysfunction of these interfaces may disrupt immune surveillance and clearance mechanisms, exacerbating cerebral inflammation and protein aggregation [97].

### Blood-brain barrier

Neuroinflammation is not a simple response to infection, toxic protein accumulation or neurodegeneration. The immune system in the CNS, at the brain-immune interfaces, and the periphery are all involved. The brain-periphery interaction in PD occurs possibly via many interfaces, including the BBB. Of note, increasing evidence points towards a link between BBB dysfunction or increased permeability and neurodegenerative diseases, including AD and PD [43]. Studies of post-mortem brain tissue [76] as well as in vivo analyses using PET tracers [2, 71] have shown a dysregulated BBB with increased permeability in PD. However, so far, no studies investigated changes to the BBB in prodromal PD patients. In post-mortem PD brain tissue, more myeloperoxidase (MPO)-positive cells were detected in areas affected by neuropathology, such as SN and caudate putamen [43]. MPO is an important lysosomal enzyme expressed in neutrophils and macrophages, especially, in response to infection [117]. Various studies found a higher proportion of neutrophils in the blood of PD patients. Additionally, the majority of studies found an elevated neutrophil-to-lymphocyte ratio (NLR) in PD patients compared to healthy controls (summarized in [101]). An elevated NLR was recently confirmed in a prodromal PD cohort [112].

While these studies are based on late-stage PD, the neutrophil count and the NLR, which are already higher in prodromal PD, represent possible changes to the BBB that could already occur prior to PD onset. It is also possible that BBB disruption is a long-term effect of elevated circulating neutrophils in PD patients and continuous accumulating damage from activated neutrophils within the brain vasculature, which, at a critical stage, can lead to BBB disruption.

### Blood-cerebrospinal fluid barrier

The choroid plexus (CP) forming the B-CSF barrier has been identified as a highly active immunogenic niche and constitutes a relevant brain-immune interface in PD. The CP consists of a monolayer of epithelial cells that secrete CSF into the ventricles and forms the B-CSF barrier, which is highly vascularized by fenestrated capillaries. The CP uniquely integrates CNS-derived signals with inputs from peripheral immune cells, functioning as an active surveillance system that selectively recruits inflammation-resolving neutrophils, monocytes and macrophages to affected brain regions during pathological states [121]. During brain inflammation, CP epithelial cells can undergo transient specialization to support immune cells, orchestrating their recruitment, survival and differentiation, while regulating tight junctions that govern the permeability of the CP brain barrier [160]. Notably, the CP undergoes modulation in response to intestinal inflammation triggered by bacterial LPS. Following gut vascular barrier disruption, the inflammatory cascade prompts CP closure, blocking access to large molecules. A genetic model where CP endothelial cells were engineered for sustained closure showed deficits in short-term memory and anxiety-like behaviors, implying a potential link between CP dysfunction and cognitive impairments [18]. Critically, CP dysregulation is a unifying mechanism in neurodegenerative diseases, thus representing a potential therapeutic target for modulating pathological brain inflammation [163]. Reduced CSF motion has been found in PD patients compared to healthy controls [111], but CP dysfunction or reduced motion in prodromal PD patients have not been investigated yet. Additionally, the exact consequences of CP impairments on microglial function are currently unknown, thus further studies are needed to investigate this complex crosstalk.

### Glymphatic system

The recently discovered glymphatic system is a brain-wide drainage system important for the clearance of metabolites and protein waste, such as amyloid beta [62]. Impaired glymphatic clearance of metabolic waste products and α-synuclein from the brain parenchyma may contribute to the accumulation of toxic protein aggregates in PD pathogenesis and development [4]. In neurodegenerative diseases, such as AD and PD, as well as during aging, it is possible that glymphatic dysfunctions aggravate inflammation by suppressing cytokine clearance and inducing microglial activation, while neuroinflammation impairs glymphatic functions and further exacerbates the inflammatory response [27, 28, 99]. Evidently, these molecular mechanisms mutually influence each other, establishing a neuroinflammatory feedback loop possibly supporting neurodegenerative processes. Various studies showed impaired glymphatic system activity in PD patients. These studies investigated differences between PD cases and healthy controls using magnetic resonance imaging (MRI), i.e., diffusion tensor imaging, that determines the diffusivity along the perivascular space as a measure of glymphatic activity and found significantly lower glymphatic activity in patients [4, 87, 96]. One study reported a correlation between glymphatic dysfunction and the severity of motor and cognitive dysfunction. So far, no studies investigated glymphatic changes in prodromal PD. However, one study reported no differences in glymphatic activity between early-stage and late PD [87], but here, only a few patients were included.

The glymphatic system activity is significantly higher during sleep and it is important for clearing out metabolic and protein waste build-up during wakefulness [159]. Interestingly, the activity of the glymphatic system decreases with aging, and the clearance of beta-amyloid is impaired by 40% in old mice [73]. All these drivers of the glymphatic system activity were originally described in murine in vivo models, but were recently confirmed in human studies [80]. The sleep-wake cycle is controlled by the LC and norepinephrine, which support the activity of the glymphatic system during sleep [50]. Interestingly, it is hypothesized that α-synuclein inclusions found in the LC area also contribute to sleeping disorders seen in prodromal PD as well as impaired glymphatic activity [16, 161]. A recent study found that already one night of sleep deprivation could increase the burden of amyloid beta [125]. Until now, only one study investigated α-synuclein in the human brain and found that one night of sleep deprivation in 30–60-year-old individuals significantly increased α-synuclein levels by 30–50% compared to individuals with a night of normal sleep [56]. It is tempting to hypothesize that impaired glymphatic system activity contributes to prodromal and pre-clinical PD, as a large proportion of these patients suffer from sleep behaviour disorders, such as insomnia, RBD and restless leg syndrome. Whether impaired glymphatic system clearance and sleep disorders are connected and whether these could determine the risk of PD warrants further investigations (See Table 1. Outstanding questions).

### Meningeal lymphatic system

The discovery of meningeal lymphatic vessels (MLVs) revealed another concept of lymphatic drainage in and out of the brain and was shown to play an important role in CNS waste clearance. MLVs drain macromolecules, such as α-synuclein, antigens and even immune cells trafficking from the CSF and interstitial fluid into cervical and deep cervical lymph nodes [27]. Few studies investigated this newly discovered brain-wide lymphatic system in relation to PD. However, a recent study found that idiopathic PD patients exhibit lower MLV outflow and reduced perfusions in deep cervical lymph nodes [30]. Blocking the MLVs led to an increase of PD symptoms in A53 T α-synuclein mutant mice [165]. Of note, similarly to other tissues, lymphatic drainage in the brain also declines with age [27]. Interestingly, microglia responses are directly affected by impaired brain drainage. In the 5xFAD mouse model of AD, which had their MLVs ablated by laser, exacerbated microgliosis together with plaque deposition and neurovascular alterations was observed. Interestingly, microglial cells from the mice with ablated MLVs expressed lower levels of homeostatic microglial genes essential for their housekeeper function and increased the expression of the typical disease-associated microglia (DAM) signature, which may suggest that the DAM-like phenotype observed in aging may be caused by impaired lymphatic drainage of the brain [28]. These studies raise the question of whether microglia can mediate the changes observed in models of reduced glymphatic [99] and lymphatic flow [28]. So far, no studies exploring the glymphatic and lymphatic systems have been conducted in preclinical PD patients. By contrast, such investigations would be crucial as PD therapies could aim at increasing the drainage of the brain via these systems or they may be used to deliver PD-relevant therapeutics.

## Models for studying prodromal PD

Animal models are essential for investigating changes taking place in vivo and for developing and testing drugs. Various PD models were created focussing on dopaminergic neuronal loss, induced either by chemical exposure or by genetic modification or by combining the two approaches [10]. With the increasing focus on the prodromal stage, animal models have been created with the purpose of investigating the disease progression and for testing disease-modifying drugs (Table 2). For more information on animal models for prodromal PD see Taguchi et al. [141].

**Table 2:** Examples of animal models used to study prodromal PD

Toxic or α-synuclein models with a prodromal phenotype		
Model	Summary	Reference
C57BL/6 mice with bilateral intranasal infusion of 1 mg/nostril of 1-methyl-4-phenyl-1,2,3,6-tetrahydropyridine (MPTP)	Moderate loss of nigral neurons, moderate loss of tyrosine hydroxylase (TH) in olfactory bulb, striatum and SN. Olfactory impairment and memory deficits.	[115]
C57BL/6 mice infused with MPTP 1 mg/nostril/day for 4 consecutive days	At 7 and 28 days post last infusion, 40–60% reduction in TH observed in striatum and 25–30% less in SN. Increased astrogliosis was detected.	[148]
Bilateral intranasal infusion of MPTP in 6–8 weeks C57BL/6 male mice. Tested 11,30,40 days post injection (dpi)	Induced non-motor symptoms, such as impaired olfactory recognition, anxiety-like behaviour and impaired social consolidation. Microgliosis and astrogliosis were induced with upregulation of IL-17 A in SN and striatum.	[107]
Injection of α-synuclein preformed fibrils into the duodenum of bacterial artificial genome rats	2–4 months post-injection α-synuclein propagated from duodenum to dorsal motor nucleus of the vagus to the LC to SN in the midbrain.	[150]
Injection of α-synuclein preformed fibrils into the duodenum and pyloric muscularis layer of B6;129X1-SncaTm1Rosl/J and C57BL/6 J mice with truncal vagotomy	Spread to dorsal motor nucleus, hindbrain, LC and SN with loss of dopaminergic neurons with both motor and non-motor symptoms. After vagotomy and α-synuclein deficiency, the gut-to-brain spread was prevented.	[70]
Genetic models with a prodromal phenotype		
Model	Summary	Reference
LRRK2 R14441 C KI mice	Impairments in fine motor tasks, olfaction and gait in 24 months old mice.	[45] See overview of LRRK2 mouse models in [20]
Exhaustive exercise of Parkin^_^/^_^ and PINK1^_^/^_^ mice. Parkin^_^;^Mutator^ mice	Prominent inflammatory phenotype observed with accumulation of mitochondrial DNA mutations. STING and type I interferon-mediated inflammation.	[128]
*PARK7*/DJ-1 KO mouse with i.p. LPS injection for 6 and 24 hours	Hyporesponsive microglia phenotype in DJ-1 KO mice compared to WT mice after 6 and 24-hour treatment with LPS in vivo. Downregulation of inflammatory response genes in microglia from DJ-1 KO mice. Higher ROS levels in microglia and BMDMs from 4 months old male DJ-1 KO mice compared to WT. Higher cytokine expression in microglia from 13 months old male DJ-1 KO mice treated with LPS compared to corresponding WT mice.	[84]
Thy1-aSyn (line 61) mice Human wildtype α-synuclein overexpression under the Thy-1 promoter	Loss of striatal dopamine and motor dysfunctions at 14 months of age (40% loss). Colonic dysfunction and olfactory impairment at 4 to 5 months, disrupted circadian rhythm at 3 months, which progressed until 12 months of age. Microglia activation occurs at 1 month of age in striatum and increased levels of TNFα and at 5–6 months in SN. Increased levels of serum TNFα observed at 5–6 months. TLR4 and TLR8 increased in SN at 5–6 months and TLR2 increased in SN at 14 months of age.	[23] [157]

### Toxic or α-synuclein models

Inducible PD murine models have been largely used for investigating the corresponding disease processes. These classical acute models are mainly obtained by the systemic or intracranial injection of either neurotoxins structurally resembling to some herbicides and pesticides, such as 1-methyl-4-phenyl-1,2,3,6-tetrahydropyridine (MPTP), or α-synuclein preformed fibrils, respectively. The intranasal infusion of MPTP or the injection of α-synuclein preformed fibrils into the duodenum enable to recapitulate some of the early-stage symptoms of PD, including non-motor signs, therefore representing relevant models for investigating prodromal PD (Table 2).

### Genetic models

The large majority of the mouse models carrying PD mutations do not have significant neurodegeneration and often few PD-like symptoms. However, they represent essential tools to study the prodromal stages of PD, as they separately assess the contribution of disease-inducing factors such as oxidative stress (DJ-1), mitochondrial dysfunction (PINK1, Parkin, Miro1) and lysosomal impairments (GBA) [123]. Our studies of microglia from DJ-1 KO mice and in DJ-1 mutant human induced pluripotent stem cell (iPSC)-derived microglia uncovered a distinct hyporesponsive phenotype when treated with LPS [84]. The response of microglia and BMDMs from DJ-1 KO mice compared to WT mice was characterized by downregulation of inflammatory response genes and a less prominent amoeboid morphology (Table 2). Interestingly, we also detected a decrease in MHC class II in microglia from DJ-1 KO mice, supporting results from RBD patient blood monocytic cells [36, 151]. The main knowledge about the factors leading to neurodegeneration comes from animal studies and especially from studies looking at genetic PD (Table 2).

## Anti-inflammatory treatments against PD development and progression

The early indications that the immune system was deregulated and over-activated in PD patients led to the treatment of experimental animal models of PD with non-steroidal anti-inflammatory drugs (NSAIDs) [3]. NSAIDs are scavengers of hydroxyl radicals and nitric oxide and can inhibit NFκB and cyclooxygenase (COX) 1 and COX2, which are key enzymes in prostaglandin synthesis. Microglia are the main prostaglandin producer in the brain [146]. Not surprisingly, NSAIDs had neuroprotective functions in PD animal models [140, 143]. Later on, large patient cohorts revealed that the use of non-aspirin NSAIDs in humans lowered their risk of developing PD [3, 114]. However, other studies could not show any benefits with NSAIDs and risk or progression of PD. These opposing findings can probably be explained by the very broad anti-inflammatory effect of these drugs, which do not have a beneficial therapeutic effect *per se* given the lack of specificity and timing. Myeloid specific drugs, such as NLRP3 inhibitors, were proven to inhibit dopaminergic neurodegeneration in a mouse model of PD [46]. Clinical trials are now investigating the effect in PD patients using a potent, selective, and CNS-penetrant NLRP3 inhibitor, NT-0796, with so far positive results from a phase Ib/IIa trial. It is possible that NLRP3 inhibitors given at an early time point of the disease are more likely to be beneficial, which would be an interesting time point to investigate. In addition to non-motor symptoms making individuals clinically at risk for developing PD, some individuals are also genetically at risk for a PD diagnosis later in life. The most common pathogenic genetic variants found in *LRRK2*, encoding LRRK kinase 2, and *GBA1*, encoding the enzyme β-glucocerebrosidase, confer a risk of 43% and 19% of developing PD by the age of 80, respectively [131]. The myeloid-specific LRRK2 kinase inhibitor recently proved safe and efficient in preclinical and clinical trial phase I and Ib [64]. In 2024, a new mutation causing late-onset PD was discovered in the *RAB32* gene, which codes for a GTPase involved in mitochondrial transport and binding to LRRK2, which stimulates its kinase activity [48, 57]. Interestingly, similarly to *LRRK2*, *RAB32* is especially highly expressed in microglia and macrophages playing a critical role in response to bacterial infections [21, 83, 147]. Further investigations of the function of this protein in microglia, but also in monocytes and macrophages in prodromal PD, would be noteworthy as well as investigating whether kinase inhibitors would work on these patients. Another example of targeting patients with common pathogenic genetic variants is represented by trials including patients with GBA1 variants, which make the enzyme β-glucocerebrosidase dysfunctional. Interestingly, increasing the CSF levels of β-glucocerebrosidase using the repurposed mucolytic drug Ambroxol was safe in a phase II clinical trial [100]. A randomized controlled trial is following up to study whether Ambroxol can modify the disease course in patients with GBA1 variants [126].

These ongoing clinical trials using inhibitors or inducers of dysregulated immune-related functions on specific patients underline the importance of focusing on immune changes in prodromal PD and stratifying patients early for the use of disease-modifying therapeutics. For more information on ongoing prevention therapies and clinical trials in preclinical PD see Crotty and colleagues [26].

## Improving the detection of prodromal PD and modifiable risk factors

One of the many challenges when investigating PD is the wide variety of non-motor symptoms, which leads to low diagnostic accuracy during its prodromal and preclinical stages. The large majority of studies and trials in prodromal PD patients only included polysomnography-positive RBD patients, as these represent the largest group with around 70–80% of them converting to diagnostic PD. A recent initiative aims at identifying clinical, genetic, serological, CSF, and imaging biomarkers of PD progression. The Parkinson’s Progression Markers Initiative is currently ongoing, collecting data from multiple centres worldwide including de novo PD patients and healthy controls [127]. The patient data collected from this initiative paved the way for α-synuclein seeding assay enabling to detect individuals with synucleinopathies from serum samples. Additionally, they could distinguish individuals with PD versus MSA by the seed conformation of α-synuclein from the blood [106]. Furthermore, the α-synuclein seed amplification assay was used to detect α-synuclein seeds in CSF of RBD patients just a few months after their first symptoms, which suggests that α-synuclein is present in the very early prodromal phases of this RBD patient group [25].

One of the newly added markers for prodromal PD is physical inactivity [53]. It increases the risk of developing PD with a likelihood ratio of 1.3, where low physical activity is defined as less than 1 hour per week of activity raising the heart rate and causing sweating [35, 53]. Evidences from murine in vivo studies show that voluntary exercise decreases the activation of microglia and astrocytes in aged mice [51]. Furthermore, it showed a higher clearance of amyloid beta via the glymphatic system and a significantly improved cognitive function. Another report showed no difference in astrocyte activation, but a higher glymphatic activity in exercised mice compared to sedentary mice [155]. Whether a significant increase of α-synuclein clearance also occurs post-exercise in humans needs further investigation, but recent studies of the effect of exercise in prodromal PD patients are promising. Interestingly, investigations of the effect of physical activity on prodromal features of PD found a reduced prevalence of constipation, excessive daytime sleepiness, depression and bodily pain in older individuals who are more physically active in midlife [60]. This work is consistent with previous studies showing that high levels of physical activity reduce the severity of motor symptoms and constipation in early PD [149]. A clinical trial, NCT06193252, recruiting prodromal PD patients started in 2024, will investigate if regular exercise improves symptoms and disease progression. Furthermore, various studies also investigated how exercise is important for improved sleep in PD [67, 124].

## Outlook

Neuroimaging studies have provided valuable insights into microglial activation patterns, which can be seen in RBD positive PD patients in the prodromal stage and at disease diagnosis. Further studies in other prodromal PD patient groups are needed to confirm if early microglia activation is a general feature of prodromal PD or mainly of RBD patients. However, using microgliosis as a putative biomarker for diagnosis and progression of PD is a promising field, but larger and systematic investigations taking into account several parameters, including microglial substates and diversity, are required. In addition, further cell specific PET tracers are needed to elucidate which cells and when they contribute to neuroinflammation in the disease process.

Targeting phenotypic acquisitions of microglia and immune dysfunctions in patients at risk of PD could represent promising preventive measures and novel therapeutic strategies for early intervention and disease modification. To this aim, the use of longitudinal patient cohorts and improved mouse models will be key to investigate what drives the disease progression in the prodromal phases of PD.

## Data Availability

No datasets were generated or analysed during the current study.

## Abbreviations

AD: Alzheimer’s disease
BBB: Blood brain barrier
BMDM: Bone marrow-derived macrophage
CSF: Cerebrospinal fluid
CNS: Central nervous system
DAM: Disease associated microglia
DPI: Days post injection
GBA: Glucosylceramidase beta
HC: Hippocampus
HSV-1: Herpes simplex virus 1
IBD: Inflammatory bowel disease
LC: Locus coeruleus
LBP: Lipopolysaccharide binding protein
LPS: Lipopolysaccharide
MRI: Magnetic resonance imaging
MLV: Meningeal lymphatic vessel
MPTP: 1-methyl-4-phenyl-1,2,3,6-tetrahydropyridine
MPO: Myeloperoxidase
MSA: Multiple system atrophy
NLR: Neutrophil to lymphocyte ratio
PET: Positron emission tomography
PD: Parkinson’s disease
REM: Rapid eye movement
RBD: REM Sleep behaviour disorder
SARS-CoV-2: Severe Acute Respiratory Syndrome Coronavirus 2
SN: Substantia nigra
SPECT: Single-photon emission computed tomography
TH: Tyrosine hydroxylase
TLR: Toll like receptor
TSPO: Translocator protein
